# Location of DNA damage by charge exchanging repair enzymes: effects of cooperativity on location time

**DOI:** 10.1186/1742-4682-2-15

**Published:** 2005-04-08

**Authors:** Kasper Astrup Eriksen

**Affiliations:** 1 Department of Theoretical Physics, Lund University, Sölvegatan 14A, SE-223 62 Lund, Sweden

## Abstract

**Background:**

How DNA repair enzymes find the relatively rare sites of damage is not known in great detail. Recent experiments and molecular data suggest that individual repair enzymes do *not *work independently of each other, but interact with each other through charges exchanged along the DNA. A damaged site in the DNA hinders this exchange. The hypothesis is that the charge exchange quickly liberates the repair enzymes from error-free stretches of DNA. In this way, the sites of damage are located more quickly; but how much more quickly is not known, nor is it known whether the charge exchange mechanism has other observable consequences.

**Results:**

Here the size of the speed-up gained from this charge exchange mechanism is calculated and the characteristic length and time scales are identified. In particular, for *Escherichia coli*, I estimate the speed-up is 50000/*N*, where *N *is the number of repair enzymes participating in the charge exchange mechanism. Even though *N *is not exactly known, a speed-up of order 10 is not entirely unreasonable. Furthermore, upon over expression of all the repair enzymes, the location time only varies as *N*^-1/2 ^and not as 1/*N*.

**Conclusion:**

The revolutionary hypothesis that DNA repair enzymes use charge exchange along DNA to locate damaged sites more efficiently is actually sound from a purely theoretical point of view. Furthermore, the predicted collective behavior of the location time is important in assessing the impact of stress-ful and radioactive environments on individual cell mutation rates.

## Background

Bases in DNA suffer damage both from normal cellular functions such as metabolism and from external oxidative stress and radiation. Naturally the cell has several lines of defense against direct lesions and ensuing mutagenic mispairings [1-3]. Oxidation of the base guanine (G) often results in the formation of 8-oxo-G (7,8-dihydro-8-oxo-2'-deoxyguanosine) [4]. During replication, 8-oxo-G can pair both with cytosine (C) and adenine (A) [5]. Following another round of replication, the 8-oxo-G:A pairs give rise to G:C to T:A mutations (if not corrected). In *Escherichia coli*, 8-oxo-G:C pairs are detected by the DNA glycosylase MutM (formamidopyrimidine glycosylase), which subsequently excises the 8-oxo-G from the DNA leaving an abasic site where the strand is nicked at both the 3' and 5' ends [6]. The abasic site is further processed by the base excision pathway (BER), eventually leading to the insertion of a G opposite the remaining C. The action of MutM brings the number of adenines A misincorporated opposite 8-oxo-G during replication down to around one per one million bases, even in cells challenged by *H*_2_*O*_2 _[7,8]. In *E. coli *the 8-oxo-G:A pairs are detected by another DNA glycosylase, MutY [9,10], which excises the mispaired adenine A leaving an abasic site. The abasic site opposite the unpaired 8-oxo-G is further processed by the BER pathway, resulting in an 8-oxo-G:C pair. If on the other hand a G in the dGTP pool is initially oxidized and subsequently incorporated opposite an A during replication, the action of MutY increases the mutagenic conversion rate of T:A to G:C. Experimentally, this is seen in strains lacking the MutT enzyme [11] responsible for the hydrolysis of 8-oxo-dGTP to 8-oxo-dGMP [12].

Both the biochemical and mechanistic functions of the excision process and the specific recognition of the base to be excised have been unraveled for many DNA glycosylases [7,13,14]. The main step is flipping the base to be excised out of DNA and into a cleft in the DNA glycosylase. This extra-helical state is associated with a kinking of the DNA through an angle of 60°–80° depending on the particular DNA glycosylase. Even though questions still remain to be answered in this area, the main challenge is to understand how the mismatched oxidized base pair is located among all the normal, correctly paired ones [13]. Direct visualization using atomic force microscopy (AFM) reveals that the human 8-oxo-G DNA glycosylase hOGGl and the *E. coli *DNA glycosylase AlkA kink error-free DNA in the same way they kink DNA during the excision of a damaged base [15]. It is thus likely that some DNA glycosylases also flip correctly-paired bases into the active site cleft during their search for excision targets [16]. Furthermore, in vitro studies indicate that some DNA glycosylases including MutY move along the DNA while scanning its integrity [17].

Until recently it was more or less implicitly assumed [16] that the individual DNA glycosylases locate damaged DNA sites independently of each other. However, a bold new hypothesis suggests a certain sub-class of DNA glycosylases might cooperate in order to locate the damaged sites more quickly [18]. This sub-class is defined by the presence of an evolutionarily well-conserved [4Fe-4S]^2+ ^cluster and includes MutY and endonuclease III, but not MutM or AlkA [1]. Endonuclease III recognizes oxidized and ring-fragmented pyrimidines, while AlkA recognizes a wide spectrum of alkanated base adducts (both alkanated pyrimidines and purines). Thus the [4Fe-4S] cluster is not obviously associated with the recognition of specific substrates. Initial investigations suggested that the cluster is not redox active under physiological conditions [19]. This led to the speculation that the [4Fe-4S]^2+ ^cluster might be a rare example of a metal cluster with a purely structural role [20]. However, it was recently shown in vitro that upon binding of MutY to DNA, an electron is injected into the DNA and the [4Fe-4S] cluster is involved in this redox reaction, presumably changing its oxidation level from 2+ to 3+ [18]. The authors then went on to hypothesize that the MutY enzymes communicate through currents in the DNA and in this way accelerate error the location process. An error-free stretch of DNA is a good conductor, while a defective base pair introduces a huge resistance [21]; if a MutY enzyme receives an electron from an upstream MutY enzyme, the stretch of DNA ahead of it is thus error-free. Presumably the electron received destabilizes the binding of MutY to this error-free stretch of DNA by changing the oxidation level of the [4Fe-4S]^3+ ^cluster back to 2+. Thus, the net-effect of the charge exchange is rapid detachment of the MutY molecules from error-free DNA, followed by binding and scanning elsewhere. Intuitively, this fast detachment of MutY enzymes from error-free stretches of DNA speeds up the location of damaged base pairs. It should perhaps be emphasized here that the proposed mechanism is speculative and has not yet been firmly verified experimentally. Nevertheless it is of interest to estimate the extent of the potential speed-up and to consider whether there are other biologically relevant and experimentally testable consequences of the proposed charge exchange mechanism. As discussed in detail below there are two relevant time scales in the proposed process. The first, *τ*, is the time it takes to realize that a stretch of DNA is error-free, i.e. *τ *is the time from attachment of a MutY enzyme attach to an error-free piece of DNA until detachment and binding to another site. The second, *T*, is the average time it takes to locate a damaged base pair by slowly scanning the DNA, without utilizing the charge exchange mechanism. In this paper, I show that the time it takes for MutY to locate a damaged base pair is roughly , corresponding to a speed-up of . This expression for the speed-up remains valid in the presence of many other kinds of charge exchanging repair enzymes. However, in this case, *T *is the average time that it takes for *any *repair enzyme to locate the error by scanning alone. Secondly, I also point out that the charge exchange mechanism alters the response to over-expressed repair enzymes. As the total number of repair enzymes is increased the efficiency of the charge exchange mechanism decreases. In this way, doubling the number of repair enzymes only shortens the location time by 30%, not by 50% as in the independent scanning scenario. The gain relative to the independent scanning scenario is thus smaller.

## Results

### Model

**Figure 1:**
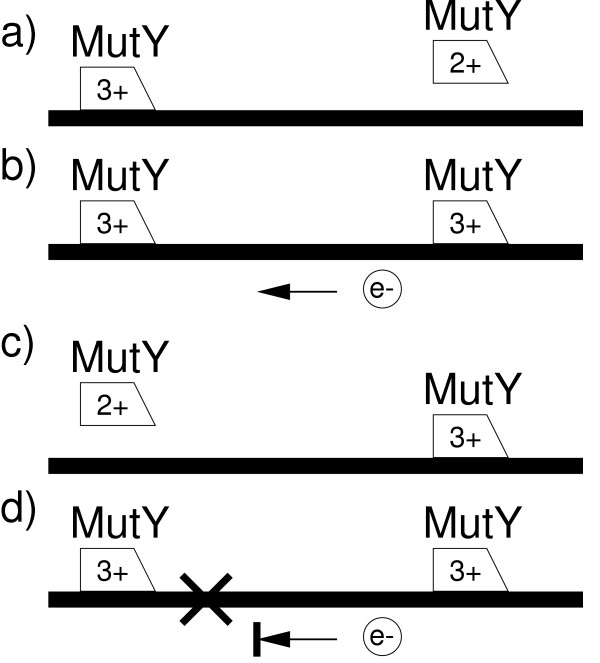
**The model**. a) The left MutY repair enzyme is bound to DNA and slowly progress to the right while it scans the integrity of base pairing. The [4Fe-4S] cluster in MutY is in a 3+ charge configuration when bound to DNA, but a 2+ configuration when not bound (right MutY). b) Upon binding to DNA the right MutY enzyme emits an electron into the DNA and changes the charge of its [4Fe-4S] cluster from 2+ to 3+. c) If the DNA is free of errors, the emitted electron travels along the DNA until it reaches the left MutY enzyme. Here the electron changes the charge of the [4Fe-4S] cluster to 2+ and thus destabilizes the DNA binding of this MutY enzyme. The left MutY enzyme then attaches to and scans a different section of DNA that is more likely to contain an error, d) If on the other hand the DNA segment between the two MutY enzymes contains an error, the electron never reaches the left MutY enzyme, which then keeps scanning the DNA until it reaches and fixes the error. The charge exchange thus selectively frees up resources from error free patches of DNA. The model is also described in [18,24].

The model is presented in Figure 1. The repair enzyme MutY contains an evolutionarily well-conserved [4Fe-4S] cluster that is suspected to change its charge configuration from 2+ to 3+ upon binding to DNA [18]. Binding is thus associated with the emission of an electron into the DNA, while upon receipt of an electron from DNA the MutY-DNA binding complex is destabilized. As only error-free stretches of DNA are able to transport the electron from a MutY enzyme to a neighboring one [21], this charge exchange enables MutY to liberate scanning resources quickly from error-free stretches of DNA [18]. To make the argument and calculations as transparent as possible, I first consider the scenario where only MutY enzymes participate in the charge exchange. However, in real cells, many different kinds of repair enzymes each are expected to participate in the charge exchange, each specialized for fixing a specific kind of damage. This more general scenario is the focus of the next section. Finally, the effect of a finite scan length before MutY spontaneously detaches from the DNA is considered.

#### Only MutY participates in the charge exchange

How long is the time *t*_location _that elapses between damage to a base pair and detection of the error by MutY? The faulty base pair is either located by a MutY enzyme that happened to be bound to DNA downstream of the error at the time of damage or by one that subsequently binds to DNA downstream of the error and then scans the DNA until it finds the damaged base pair. In the regime where the charge exchange mechanism markedly accelerates the error location, the second mechanism dominates. Let *t*_location _denote the typical location time and *v *the scanning velocity of MutY. The rate at which a MutY enzyme randomly docks onto a specific base pair and starts scanning is denoted by *k*. The probability that a MutY enzyme lands within a distance of *vt*_location _of the error in the time interval *t*_location _can be estimated as . This probability is of order 1, since in the time *t*_location _a MutY enzyme typically arrives at the faulty base pair. Thus



A more detailed derivation yields the same result apart from a factor of 1.3 (See additional file: MutY_detailed_derivation.pdf). However this factor is not reliable as no model fully incorporates all biological processes. Consequently I have made no attempt to keep track of such factors in the following argument. The average docking rate *k *can be expressed as



where *τ *is the time between two successive binding events for a single MutY enzyme. *N*^MutY ^is the total number of MutY enzymes. *L *is the total number of base pairs in DNA. *T *= *L*/*v*/*N*^MutY ^is the time it takes for the MutY enzymes to scan all the bases of DNA once. It is here assumed that all the MutY enzymes belong to a single freely-exchanging pool and that MutY is equally able to bind to all *L *base pairs of DNA. Considering that DNA is folded into chromatin superstructures, this is probably not true, but as a first rough estimate it suffices. In terms of *T *and *τ *the location time is (combining Eqs. (1) and (2))



In the traditional scenario where the MutY enzymes scan the DNA independently to locate the mispaired sites, 1/*v *is the time it takes a single MutY enzyme to check the integrity of one base pair. According to standard Poisson statistics the location time without charge exchange mediated cooperation between the MutY enzymes is *L*/*v*/*N*^MutY ^= *T*. Cooperation thus gives a speed-up of approximately .

#### Many different kinds of repair enzymes

The functionally central [4Fe-4S] cluster is also present in other repair enzymes e.g. endonuclease III. Very likely these repair enzymes are also are able to inject charges into DNA and participate in electrical scanning. Consequently these charge sensitive repair enzymes are also 'attracted' to the damaged DNA pair in exactly the same way as MutY. Thus in the above model and calculation, 'MutY' can be replaced by 'any repair enzyme participating in DNA mediated charge transport' (repair enzyme). Likewise the calculated location time *t*_location _is the time before the first repair enzyme locates the damaged site and *T *is the average time it takes for any repair enzyme to find the site without using currents. Here I have implicitly assumed that both the scan velocity *v *and the time *τ *between successive binding events are of the same orders of magnitude for all repair enzymes i.e. MutY is a typical repair enzyme. Biologically, the time *t*_location _is not the most relevant one as the first repair enzyme that arrives at the damaged base pair is probably unable to fix the damage. On average the first MutY enzyme is the *N*/*N*^MutY ^repair enzyme to arrive at the damaged site. Thus the MutY location time



Here *N *is the total number of repair enzymes. *N*/*N*^MutY ^can also be expressed as *T*^MutY^/*T*, where *T*^MutY ^is the time it takes for the MutY enzymes to locate the site by scanning alone. Using Eq. (3) the MutY location time is



The speed-up relative to the independent scanning of the genome is thus again . However this time, *T*, is the time it takes for any repair enzyme to locate the damage.

#### Finite scan length

MutY is known to detach from DNA spontaneously after scanning in the order of 100 base pairs (bp) [17]. In order to estimate the resulting effect, if any, on the MutY location time, Eq. (5) is derived in a slightly different manner. The MutY enzyme that eventually locates the damage typically docks on to DNA within a distance Δ from the faulty base pair. Δ fulfills two constrains. First it is less than 100 bp in order to avoid spontaneous detachment of the MutY from the DNA before it has scanned the damaged site. Secondly it is so small that the probability that another repair enzyme will dock on to the DNA in front of MutY is less than 1. As the distance from MutY to the error is roughly Δ and the time it takes to scan the Δ intervening bases is Δ/*v*, the latter probability is approximately *k*ΔΔ/*v*. Thus Δ ≤ . In terms of Δ, the MutY location time  is determined as above by setting the probability that a MutY enzyme docks within a distance Δ in the time interval  equal to 1 i.e. *k*^MutY ^ Δ = 1 or



*T*^MutY ^= *N*^MutY ^/ *L*/*v *= (*k*^MutY^*vτ*)^-1 ^is the location time in the scenario, where the MutY enzymes act independently of each other. The length



is the distance over which the charge exchange typically takes place. In the section 'Many different repair enzymes', *l *was the average distance between two repair enzymes *vT*. However, in vivo, other factors might limit *l *and the expression



is the most general expression for the reduction in location time due to the charge exchange mechanism: *T*^MutY ^/ .

### Estimating order of magnitude

Since no experimental data exist for *τ *and *l*, the efficiency of the charge exchange mechanism, Eq. (8), must be estimated. The numerator Δ is the smallest of the maximal scan length 100 bp and the docking distance . I assume  ≤ 100 bp, with equality as the most likely option, as anything else seems inefficient. The distance *l *is estimated as the average distance between the repair enzymes *vT *= *L*/*N*. With these approximations the reduction is  ≥ *vT*/100 bp = 5·10^4^/*N*, where *N *is the total number of repair enzymes with a charge exchange mechanism similar to MutY. I have assumed that the length of *E. coli's *DNA, *L*, is 5·10^6 ^base pairs. Unfortunately *N *is unknown. The numbers of the two [4Fe-4S]^2+^-containing repair enzymes, MutY and endonuclease III, are estimated to be 30 and 500 respectively and the number of MutM repair enzymes is estimated at 400 [22]. The primary target of MutM, 8-oxo-G, is estimated to constitute 5% of all adducts due to oxidative damage [4]. All in all it seems reasonable that the total number of repair enzymes participating in the charge exchange mechanism is significantly smaller than 50000, and that a speed-up of order 10 is realistic. Notice this would correspond to a typical scan length that is 10 times smaller than the maximal one (100 bp) and that *l *≈ 1000 bp.

## Discussion

The implications of a proposed charge exchange mediated cooperation between repair enzymes in locating defects in single base pairs have been considered. From the theoretical point of view taken here, this mechanism is likely to speed up location by a factor of order 10 compared to the traditional scenarios in which the repair enzymes scan the genome for errors independently. In this paper the speed-up was quantified in terms of the time it takes to locate a damaged base pair *t*_location_. *t*_location _has to be considerably shorter than the replication time, which in *E. coli *is in the order of one hour. To be concrete, assume *t*_location _is 20 minutes. For the 30 MutY enzymes in *E. coli *the calculated efficiency of the charge exchange mechanism translates into a reduction in the necessary scan velocity from 125 bp/s to 13 bp/s. For comparison, the scan velocity for RNA polymerase is 50 bp/s, while for DNA polymerase it is 1000 bp/s.

In the traditional independent-scanning scenario the location time *T *is inversely proportional to the number of repair enzymes. Upon over-expression of all the repair enzymes the effective distance over which the charge exchange takes place, *l*, is reduced and the efficiency of cooperation is reduced (Eq. 8). Thus the decrease in location time  is smaller in the charge exchange scenario than in the traditional independent-scanning scenario, but *t*_location _remains shorter than *T*. Note that if only a small subclass, such as MutY, is over-expressed, the location time is still inversely related to the number of molecules. Assuming that the typical scan length *vτ *remains constant during over-expression the location time is inversely proportional to the square root of the total number of repair enzymes. The important point is not the exact square root behavior but the relative insensitivity to simultaneous over-expression of all the repair enzymes. Physiologically, oxidative and radiative environments may result in an increased expression of repair enzymes [23], so the relative insensitivity of the location time and the coupling of the effectiveness of different kinds of repair enzymes are potentially of huge importance for mutation rates in these kinds of stress full environments.

## Conclusion

I have demonstrated that the charge transport mechanism indeed offers great potential benefit for the cell. However, only further experimentation can finally confirm the charge transport mechanism, the current status of which must be dubbed speculative. Furthermore, I have pointed out that the charge transport hypothesis, if valid, has consequences for the cellular response to stress-ful environments. In addition, the model is a simple model of protein cooperativity and one might wonder if the principles underlying it could be of practical use in apparently unrelated engineering problems.

## Competing interests

The author(s) declare that they have no competing interests.

## Supplementary Material

Additional File 1 Contains a more detailed derivation of Eq. (1), keeping track of all the numerical factors. 1 page. Click here for file
