# Supplementary material for: Location of DNA damage by charge exchanging repair enzymes: effects of cooperativity on location time
Source: Theor Biol Med Model. 2005 Apr 8;2:15. doi: 10.1186/1742-4682-2-15 (PMC1142343; doi:10.1186/1742-4682-2-15)
Supplement: Additional File 1 — Contains a more detailed derivation of Eq. (1), keeping track of all the numerical factors. 1 page. [file 1742-4682-2-15-S1.pdf]

# 1 Appendix

Assume that at $t = 0$ an error arises in base pair 0. First, the probability that the error will not be detected before time $t$: $P(\text{error not found at time } t)$ is calculated. In order to detect the error a MutY enzyme has to bind next to the damaged site and then scan the DNA all the way up to and including the damaged base pair. Thus the error is undetected if no MutY enzyme binds to DNA within $v(t - s)$ base pairs of the error at any instant $s < t$ prior to $t$. The probability that no MutY enzymes with a scan direction towards the error will bind in the region $v(t - s)$ either up or downstream of the error in a small time interval $\Delta s$ around $s$ is given by the Poisson distribution

$$\exp[-kv(t - s)\Delta s]\,. \tag{1}$$

Note that the ability of MutY to bind to DNA on both sides of the erroneous site cancels the factor 0.5 arising from the fact that only half of the MutY enzymes scan towards the error. (The other half go in the opposite direction).

The probability that the error will remain undetected for a time period $t$ is the product of all the Poisson probabilities Eq. (1) for $s < t$:

$$\begin{aligned} P(\text{error not found at time } t) &= \exp[-kv \int_0^t t - s\, ds - t/T] & (2)\\ &= \exp(-kvt^2/2 - t/T)\,. & (3) \end{aligned}$$

The second term, $t/T$, covers the case where the MutY detecting the damage is pre-bound to DNA directly downstream of the damage at $t = 0$. In the main text this term was neglected as it is not important when $t_{\text{location}} \ll T$. For simplicity it is assumed that MutY scans for ever and the effect of collisions between MutY enzymes is neglected. The latter constraint is effectively equivalent to assuming that MutY enzymes scanning in a direction away from the error fall off immediately because of communication with other repair enzymes further away. The probability that the error will be detected after exactly time $t$ is then, as usual, given by $-\frac{d}{dt}P(\text{error not found at time } t)$ and the average detection time

$$\begin{aligned} t_{\text{location}} &= -\int_0^\infty t \frac{d}{dt} P(\text{error not found at time } t)\, dt \\ &= \sqrt{\pi/2}\frac{1}{\sqrt{kv}}\left[\exp(\frac{1}{2kvT^2})\text{erfc}(\frac{1}{T\sqrt{2kv}})\right]\,. & (4) \end{aligned}$$

where erfc is the complementary error function. In the limit $\tau \ll T$ i.e. $kvT^2 = T/\tau \gg 1$ Eq. (4) reduces to

$$t_{\text{location}} = \sqrt{\pi/2}\frac{1}{\sqrt{k\,v}}\,, \tag{5}$$

which is identical to Eq. 1 in the main text apart from the numerical factor $\sqrt{\pi/2}$.
